# Ferroptosis as an emerging target in rheumatoid arthritis

**DOI:** 10.3389/fimmu.2023.1260839

**Published:** 2023-10-19

**Authors:** Hui Zhao, Cheng Tang, Miao Wang, Hongfang Zhao, Yan Zhu

**Affiliations:** ^1^ The Geriatrics, Graduate School of Anhui University of Chinese Medicine, Hefei, China; ^2^ Shuguang Hospital, Shanghai University of Traditional Chinese Medicine, Shanghai, China; ^3^ The Geriatrics, The Second Affiliated Hospital of Anhui University of Chinese Medicine, Hefei, China

**Keywords:** ferroptosis, rheumatoid arthritis, lipid peroxidation, iron metabolism, emerging target

## Abstract

Rheumatoid arthritis (RA) is an autoimmune disease of unknown etiology. Due to the rise in the incidence rate of RA and the limitations of existing therapies, the search for new treatment strategies for RA has become a global focus. Ferroptosis is a novel programmed cell death characterized by iron-dependent lipid peroxidation, with distinct differences from apoptosis, autophagy, and necrosis. Under the conditions of iron accumulation and the glutathione peroxidase 4 (GPX4) activity loss, the lethal accumulation of lipid peroxide is the direct cause of ferroptosis. Ferroptosis mediates inflammation, oxidative stress, and lipid oxidative damage processes, and also participates in the occurrence and pathological progression of inflammatory joint diseases including RA. This review provides insight into the role and mechanism of ferroptosis in RA and discusses the potential and challenges of ferroptosis as a new therapeutic strategy for RA, with an effort to provide new targets for RA prevention and treatment.

## 1 Introduction

Rheumatoid arthritis (RA) is a multifactorial autoimmune disease of unknown etiology mainly characterized by synovial hyperplasia, pannus formation, as well as cartilage and bone destruction, resulting in joint pain, stiffness, and swelling. In RA pathogenesis, activated RA fibroblast-like synoviocytes (RA-FLSs) exhibit proliferative features similar to tumor cells and subsequently cause cartilage erosion, which will eventually lead to joint destruction (1, 2). RA patients initially present with the main complaint of finger and/or wrist joint pain, but as the disease progresses, RA may also involve large joints such as the knee, shoulder, and hip joints, resulting in limited joint activity, irreversible joint deformities, and even disability. Apart from joint symptoms, RA may also give rise to systemic multi-system injury and extra-articular manifestations (3). Due to the intensification of population aging, prolongation of life expectancy, and deterioration of the global environment, the morbidity of RA is ascending annually. Statistics estimate that approximately 1% of the global population suffers from RA, and the incidence rate of RA in women is three to four times higher than that in men (4, 5). The current treatment strategies for RA mainly focus on regulating immune inflammatory responses to alleviate arthritis symptoms (6). However, these existing treatment methods still have insurmountable shortcomings such as various adverse drug reactions, limited individualized treatment regimens, significant differences in treatment effectiveness, and drug resistance. Effective therapies that can delay or reverse the pathological progression of RA are urgently needed. Therefore, further elucidating the pathogenesis of RA and identifying new therapeutic targets will help develop more personalized, effective, and safe treatment approaches, thereby improving the quality of life of RA patients and reducing the socioeconomic burden of the disease.

It has been hypothesized that iron is excessively accumulated in the synovium of RA patients to perpetuate inflammation (7, 8). Ferroptosis is a type of regulatory cell death that depends on intracellular iron accumulation. The fundamental features of ferroptosis, such as iron deposition, lipid peroxidation, glutathione (GSH) depletion, and glutathione peroxidase 4 (GPX4) inactivation, have been gradually emphasized in the pathogenesis of RA (9). As a significant regulatory factor in inflammatory responses, ferroptosis has recently been reported to be strongly associated with the initiation and progression of various inflammatory arthritis including RA (10). This review provides an in-depth study of the role and mechanisms of ferroptosis in RA, discusses the potential and challenges of ferroptosis as a novel therapeutic strategy for RA, and highlights the potential of ferroptosis as a promising treatment target for RA.

## 2 Overview of ferroptosis

Cell death is a fundamental physiological process in all aspects of life. In the past, most studies focused on the diverse roles of apoptosis, pyroptosis, autophagy, and necrosis in diseases. In 2012, Dixon et al. first reported a novel form of regulated cell death with distinct morphological, biochemical, and genetic properties from the aforementioned cell death types, named “ferroptosis” (11). Ferroptosis morphologically exhibits the same features as necrosis, including loss of plasma membrane integrity, cytoplasm and organelle swelling, increased mitochondrial membrane density, reduced or absent cristae, and outer membrane rupture (12, 13). The exact pathogenesis of ferroptosis has not yet been clarified. Current studies indicate iron accumulation, lipid peroxidation, and redox system dysregulation as central biochemical events leading to ferroptosis (14). During ferroptosis, excessive accumulation of iron ions can catalyze the production of reactive oxygen species (ROS) through the Fenton reaction to promote lipid peroxidation, causing oxidative damage to the cell membrane (15). Subsequently, oxidative stress further damages the cell membrane and important intracellular biomolecules due to the disruption of antioxidant defense mechanisms, and this imbalance between oxidative damage and antioxidant defense is a crucial driver of ferroptosis (16, 17). GPX4 inactivation based on GSH depletion is a central regulator in the antioxidant defense system, and GPX4 can utilize the substrate GSH to reduce oxidation to scavenge free radicals and other harmful substances (18). Therefore, it can be assumed that the core mechanism of ferroptosis is the insufficient ability of GPX4 to scavenge peroxides and the excessive accumulation of lipid peroxides. Since the mechanisms of ferroptosis are exceptionally complex and involve multiple signaling pathways, an in-depth study of these mechanisms can provide new ideas and approaches for the treatment and prevention of ferroptosis-related diseases.

## 3 Ferroptosis mechanisms

### 3.1 Iron metabolism

**Figure 1:**
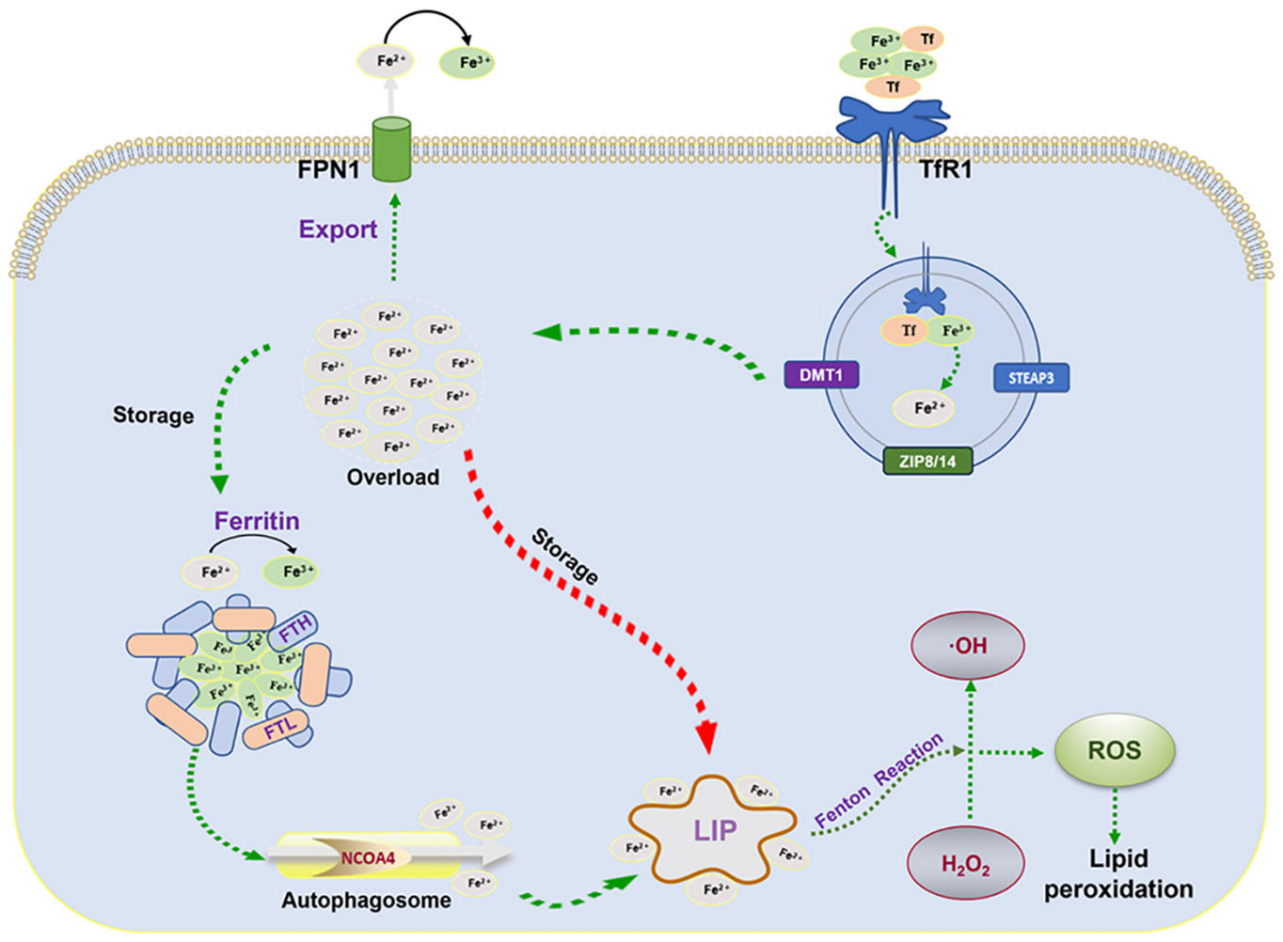
Mechanisms of iron metabolism: FPN1, iron transporter protein; TfR1, transferrin receptor 1; FTL, ferritin light chain; FTH, ferritin heavy chain; NCOA4, nuclear receptor coactivator 4; LIP, labile iron pool; ROS, reactive oxygen species; STEAP3, six-transmembrane epithelial antigen of the prostate 3; DMT1, divalent metal transporter protein 1.

As one of the abundant trace elements in the crust, iron plays an imperative role in life activities such as DNA synthesis, redox reactions, enzymatic processes, cellular respiration, and metabolism (19, 20). Therefore, precise iron metabolism is required for the homeostasis of intracellular iron ions. Ferroptosis, as a response to abnormal cellular iron metabolism, typically occurs in a state of iron overload. After the circulating iron ions (Fe^3+^) bind to transferrin (Tf) on the plasma membrane, the complex is transferred into the cell by binding to the specific transferrin receptor 1 (TfR1) and localized in the endosome (21). The acidic environment in the endosome promotes the shedding of Fe^3+^and reduces it to Fe^2+^ via the six-transmembrane epithelial antigen of prostate 3 (STEAP3), which is then exported to the cytoplasm via divalent metal transporter protein 1 (DMT1) or ZRT/IRT-like protein 8/14 (ZIP8/14). In the cytoplasm, a majority of Fe^2+^ is stored in ferritin and a small fraction of Fe^2+^ is stored in the labile iron pool (LIP). Excess Fe^2+^ is oxidized to Fe^3+^, which is transported out of the cell and re-entered into the circulation via ferroportin (FPN). Ferritin, a nanocage composed of ferritin light chain (FTL) and ferritin heavy chain (FTH), reduces Fe^2+^ to non-toxic Fe^3+^ (22). In response to the autophagic degradation by nuclear receptor coactivator 4 (NCOA4), ferritin releases active Fe^2+^ (23, 24) to catalyze lipid peroxidation through the Fenton reaction (25) (**Figure 1**).

### 3.2 Lipid peroxidation and oxidative stress

**Figure 2:**
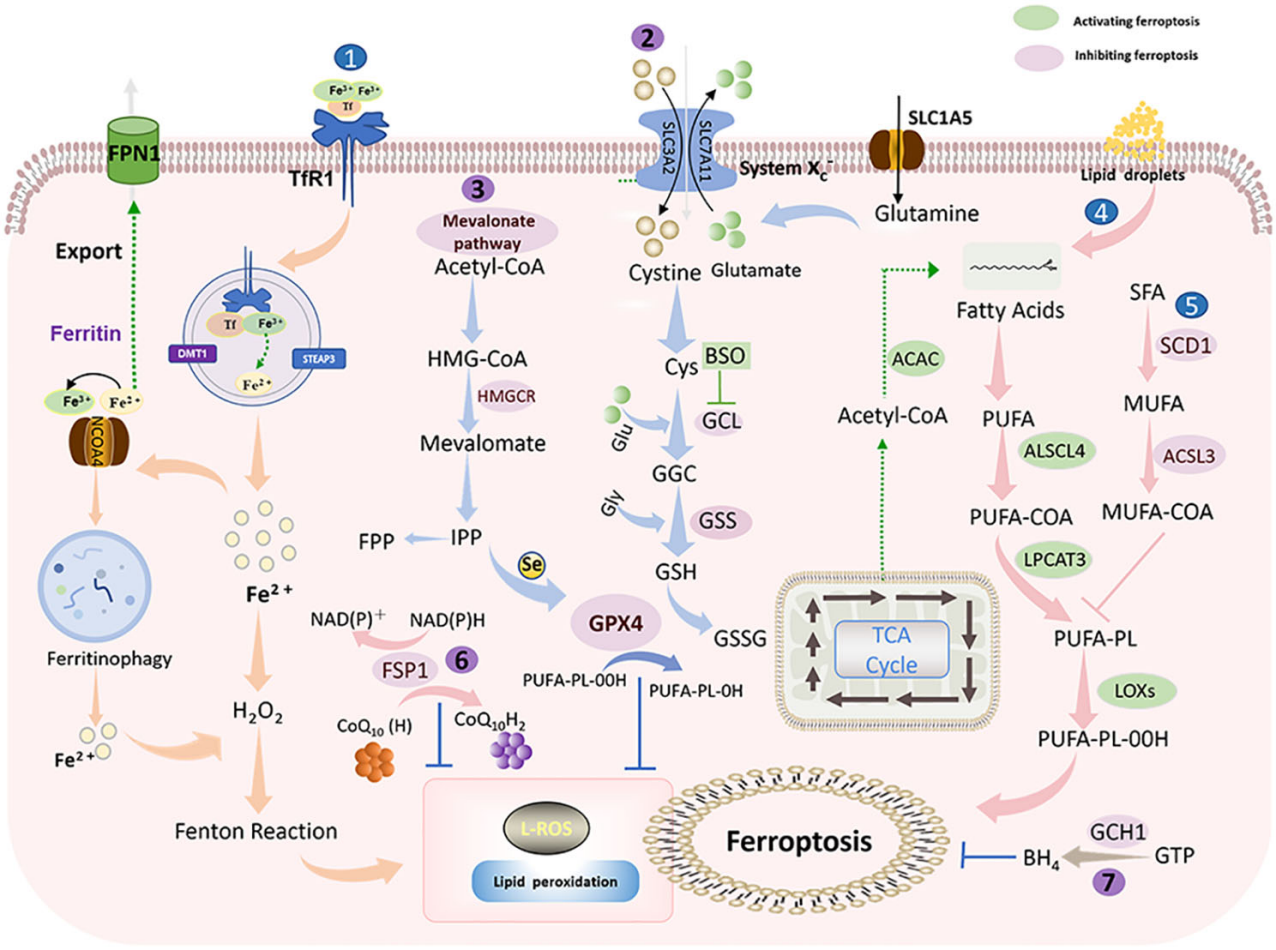
Ferroptosis mechanism: Ferroptosis is mainly caused by iron-dependent lipid peroxidation. It is mainly divided into ferroptosis promotion pathway (blue) and ferroptosis inhibition pathway (purple) (1). Iron metabolic pathway (2). Cystine/glutamate (also known as Xc-system)-GSH-GPX4 pathway: Cystine is imported into cells via the Xc-system, where it is oxidized to cysteine (Cys), followed by the synthesis of glutathione (GSH) in the presence of glutamate-cysteine ligase (GCL) and glutathione synthase (GSS). GSH is a potent reducing agent. GPX4 inhibits ferroptosis by using GSH as a reducing cofactor to reduce lipid hydroperoxides to lipid alcohols (3). Mevalonate pathway: Acetyl coenzyme A is first converted to HMG-CoA, which is then reduced to mevalonate by HMGCR, and mevalonate is converted to IPP. Finally, selenocysteine residues are added to the catalytic center of GPX4 to activate GPX4. At the same time, IPP can also produce coenzyme Q10 and then enter the FSP1 pathway (4). Lipid metabolic pathway: PUFAs are metabolized by ACSL4 and LPCAT3 and then oxidized by lipoxygenase HMGCR, 3-hydroxy-3-methylglutaryl coenzyme A reductase; IPP, isopentenyl pyrophosphate; GPX4, glutathione peroxidase 4; SLC1A5, solute carrier family 1 member 5; GLS, glutaminase; SLC3A2, solute carrier family 3 member 2. SLC7A11, solute carrier family 7 member 11; GSH, glutathione; Xc- system: glutamate reverse transporter protein, GPX4: glutathione peroxidase 4.

Ferroptosis is ultimately driven by ROS-mediated lipid peroxidation. Polyunsaturated fatty acids (PUFAs), represented by arachidonic acid (AA) and adrenaline (AdA), are most susceptible to peroxidation (15). Free intracellular PUFAs form esterification products through the binding of coenzyme A (CoA) and acyl-coenzyme A synthase long-chain family member 4 (ACSL4), which are subsequently transported to phospholipid-like lipids (PL) of the cell membrane via lysophosphatidylcholine acyltransferase 3 (LPCAT3) (26, 27). In the presence of lipoxygenase (Lox) peroxidation, PUFA-PL is oxidized, inducing the accumulation of PL hydrogen peroxide on the plasma membrane and thus affecting membrane function (28) (**Figure 2**).

### 3.3 Antioxidant defense

Lipid peroxidation can be countered by antioxidant systems, in which the cystine/glutamate-GSH-GPX4 is the main antioxidant pathway (29). Cystine/glutamate (also known as the Xc^-^ system) is a system composed of light chain solute carrier family 7 member 11 (SLC7A11, xCT) and heavy chain solute carrier family 3 member 2 (SLC3A2). Cystine is reduced to cysteine intracellularly via Xc^-^ to participate in GSH synthesis (30). Glutathione peroxide GPX4 is a primary factor responsible for the scavenging of lipid peroxides, which can convert GSH to oxidized glutathione (GSSG) and simultaneously reduce toxic lipid peroxides to non-toxic alcohols (31). Moreover, FSP1-CoQ10-NADPH (32) and GTP-BH4 (33) are two antioxidant systems independent of GPX4 that effectively protect cells from lipid peroxidation damage.

## 4 Association between ferroptosis and RA

Recent studies have found that the basic features of ferroptosis such as iron deposition, lipid peroxidation, GSH depletion, and GPX4 inactivation are implicated in RA pathogenesis. Immune inflammatory dysregulation and intestinal microbial imbalance in RA are confirmed to be associated with ferroptosis. These findings suggest that ferroptosis is closely related to the occurrence and development of RA.

### 4.1 Disorders of iron metabolism in RA

Disturbances in iron metabolism lead to the development of RA through a variety of mechanisms, including promoting oxidative stress, inducing inflammatory responses, and impairing immune cell functions. RA patients commonly present with elevated iron metabolism indicators like serum iron and ferritin, and the concentration of iron is positively correlated with the severity of joint inflammation (34, 35). FTL, FTH, and non-characteristic resistance-associated macrophage proteins such as Nramp2 and DMT1 can also be detected in fibroblast-like synoviocytes (FLSs) and macrophages isolated from synovial tissues of RA patients (36). In addition, animal experiments have also demonstrated that intravenous iron injection can exacerbate RA synovial inflammation (37). Iron acts as an important regulatory factor in immune responses, and its metabolism is of great significance to autoimmune diseases including RA (38, 39). Iron overload has profound effects on the immune system, including suppressing the phagocytosis of monocytes and macrophages, enhancing the number and viability of suppressor T cells, impairing the proliferative capacity of Th cells, and altering the distribution of lymphocytes in different compartments of the immune system (40). Moreover, iron overload may also trigger immune inflammatory responses by activating the NF-κB signaling pathway and stimulating the secretion of inflammatory cytokines such as tumor necrosis factor-α (TNF-α), interleukin (IL)-6, and IL-β (20). In a recent mouse experiment (41), the application of iron chelators effectively reduces iron deposition and ameliorates iron overload-induced oxidative damage and immune dysfunction.

### 4.2 Lipid peroxidation and oxidative stress in RA

Oxidative stress as a major pathogenic hallmark of RA promotes the local microenvironment at the RA lesion sites, enhances abnormal synoviocytes proliferation, and exacerbates inflammatory infiltration (42, 43). Oxidative stress is the result of an imbalance between the generation of reactive oxygen species (ROS) and the antioxidant defense systems. ROS-mediated mitochondrial DNA (mtDNA) tends to be recognized as a pathogen-associated molecular pattern (PAMP) by the innate immune system, leading to the activation of multiple inflammatory pathways (44). A higher ROS concentration and a lower antioxidant potential have been observed in the plasma of patients with active RA (45). Furthermore, emerging studies have confirmed that there exists a mutually reinforcing positive feedback mechanism between oxidative stress and inflammation, where ROS and mitochondrial damage products are good activators of inflammatory responses (46, 47).

In RA, excessive ROS accumulation and sustained inflammatory activation promote synovial, vascular, and joint damage. FLSs are the main effector cells of RA inflammation. The activated FLSs recruit peripheral monocytes/macrophages to synovial tissues by releasing chemokines and inflammatory mediators (48). Moreover, RA-FLSs can secrete macrophage colony-stimulating factor (M-CSF) and receptor activator of nuclear factor κB ligand (RANKL) to promote bone destruction (49). Related studies have found (50, 51) that ROS can promote the abnormal proliferation of RA-FLSs and mediate multiple pathological processes including erosion, migration, inflammation, and joint damage. As a second messenger, ROS can upregulate hypoxia-inducible factor-1α (HIF-1α), activate the JAK3/STAT4 pathway, and promote RANKL expression (52). Moreover, Notch-1 is known to be strongly expressed in the perivascular area of synovial tissues and ROS can also induce the formation of vascular opacities by upregulating the expression of vascular endothelial growth factor (VEGF) and Notch-1 (53). Of note, ROS accumulation underlies RA, and the accumulated ROS has different effects over different cell types. For example, excessive ROS is toxic to non-FSLs and triggers ferroptosis in these cells; however, ROS accumulation has a pro-proliferative and pro-survival role in FSLs. Several studies have attempted to explore alternative methods to assess the disease activity and prognosis of RA by monitoring the levels of ROS, mtDNA, and other markers of oxidative stress (46, 54, 55). However, further studies and explorations are still needed to address the use of oxidative stress markers in the treatment of RA.

### 4.3 Antioxidant defense in RA

RA patients commonly present with lower levels of antioxidant markers such as GSH and GSH peroxidase (GSH Px) compared to healthy individuals and systemic lupus erythematosus (SLE) patients, and methotrexate treatment can notably alter the oxidative stress parameters in RA patients (56). The use of antioxidants, such as vitamin E, vitamin C, and selenium, has shown promising efficacy in alleviating RA disease conditions (57–59). These findings suggest that antioxidants and oxidative stress markers may be potential therapeutic targets for RA. However, further studies and clinical trials are required to evaluate the efficacy and safety of these potential targets.

### 4.4 Immunity and inflammation in RA

Dysregulation of the immune-inflammatory response is accepted as the predominant pathogenic mechanism of RA (5). A previous study has summed up (60) the effects of ferroptosis on immune cells in two aspects. On the one hand, ferroptosis affects the number and function of immune cells. On the other hand, ferroptotic cells can be recognized by immune cells and subsequently trigger a series of inflammatory immune responses. Thus, we report the association between ferroptosis and immune cells, especially macrophages, neutrophils, and T cells upstream of RA.

Macrophages are immune cells with antigen-presenting and phagocytic properties and have critical functions in the initiation and perpetuation of RA synovitis. Iron overload promotes the polarization of M1 macrophages and increases the levels of M1 macrophage markers (IL-6, TNF-α, and IL-1β) (61). Meanwhile, TNF-α released from M1 macrophages upregulates the expressions of acyl-CoA synthetases (ACSL3 and ACSL57) (62), promotes L-ROS accumulation, and eventually triggers ferroptosis. More significantly, M1 macrophages also elevate the ROS concentration via the nicotinamide adenine dinucleotide phosphate (NADPH) oxidase 2 (NOX2) pathway (63). Furthermore, erythrocytes phagocytized by macrophages are first catabolized to heme and then to iron under the action of heme oxygenase, and iron is deposited in RA macrophages to provide suitable conditions and environment for the occurrence of ferroptosis (64, 65).

As the first line of host defense against invading pathogens, neutrophils can be the first type of innate immune cells reaching the RA synovial joints to drive inflammation by producing chemokines, ROS, and neutrophil extracellular traps (NETs) (66). Neutrophil ferroptosis prevalently occurs in SLE patients (67), but whether neutrophil ferroptosis is associated with RA is not yet clear. Notably, there is an imbalance between oxidative stress and antioxidant defense in neutrophils of RA patients (68), which is exactly the key to driving ferroptosis. Besides, mitochondrial formyl peptides (mtNFPs) are known to induce the release of ROS from neutrophils, and significantly elevated levels of mtNFPs have been found in RA patients (69), suggesting that neutrophil ferroptosis is most likely present in RA. However, mechanisms regarding neutrophil ferroptosis in RA still require further scientific studies.

T cells are the leading inflammatory cells in RA synovial tissues. Ferroptosis is found to regulate the activity of cytotoxic T cells (CD8) and helper T cells (CD4). T cell activation is dependent on the production of cysteine (Cys) by antigen-presenting cells. Ferroptosis-related proteins, Xc^-^ anti-transport proteins, and neutral amino acid transporter proteins are available to provide T cells with the required Cys (70). An *in vitro* experiment has revealed that ferroptosis inhibitor FSP1 or GPX4 overexpression protects CD8 T cells from ferroptosis, while the utilization of GPX4 inhibitors significantly enhances the sensitivity of T cells to ferroptosis (71). Further investigation (72) suggests that T cells from RA patients can store lipid droplets, leading to excess fatty acids and thus providing substrates for lipid peroxidation and ferroptosis.

### 4.5 Intestinal microorganisms in ferroptosis

With the intensive investigation of the pathogenesis of RA, there is increasing evidence supporting the association between intestinal flora and RA. Evidence points out (73) that dysbacteriosis is likely to initiate hyperactivity of the intestinal mucosal immune system, activate both innate and adaptive immunity, and then overtake the synovial joints, a process known as the “gut-joint axis”. More crucially, the intestinal microbiota also participates in ferroptosis by facilitating oxidative stress balance and reducing ROS accumulation. Excessive oral intake of iron causes intestinal iron accumulation and, in some cases, promotes ROS accumulation and interferes with intestinal microbiota homeostasis, eventually triggering RA development (74). Free iron may play a role in inflammatory diseases such as RA through the mediation of microbial activation rather than through Fenton response and oxidative stress mechanisms, implying that iron metabolism is closely related to the regulation of microbiota and immune response (75). Further, intestinal microbiota metabolites mediate ferroptosis by regulating the transferrin-long-chain acyl coenzyme A synthase 4 (TFR-ACSL4) pathway and inducing subsequent lipid metabolism disorders and inflammatory responses, while the use of iron chelators can effectively mitigate this process (76).

## 5 Potential role of ferroptosis in RA inflammation and tissue damage

### 5.1 Ferroptosis products and derivatives can act as inflammatory mediators

Ferroptosis is an immunogenic form of cell death, and its metabolites and derivatives can also act as inflammatory mediators to further exacerbate RA inflammatory responses. Ferroptosis can induce the release of damage-associated molecular patterns (DAMPs), thereby promoting immune cell activation and enhancing inflammatory responses (77). On the one hand, DAMPs promote the maturation and antigen presentation of immune cells such as macrophages and dendritic cells to enhance immune responses. On the other hand, DAMPs are well-recognized triggers that result in the elevation of inflammatory cytokine levels and then initiate inflammatory signaling pathways by binding to corresponding receptors. The execution of ferroptosis is driven by lipid peroxidation. The main products of lipid peroxidation derivatives, such as 4-hydroxynonenal (4-HNE) and malondialdehyde (MDA), may lead to cartilage degeneration and subchondral bone remodeling, and 4-HNE has also been demonstrated to activate inflammatory signaling pathways (78).

**Figure 3:**
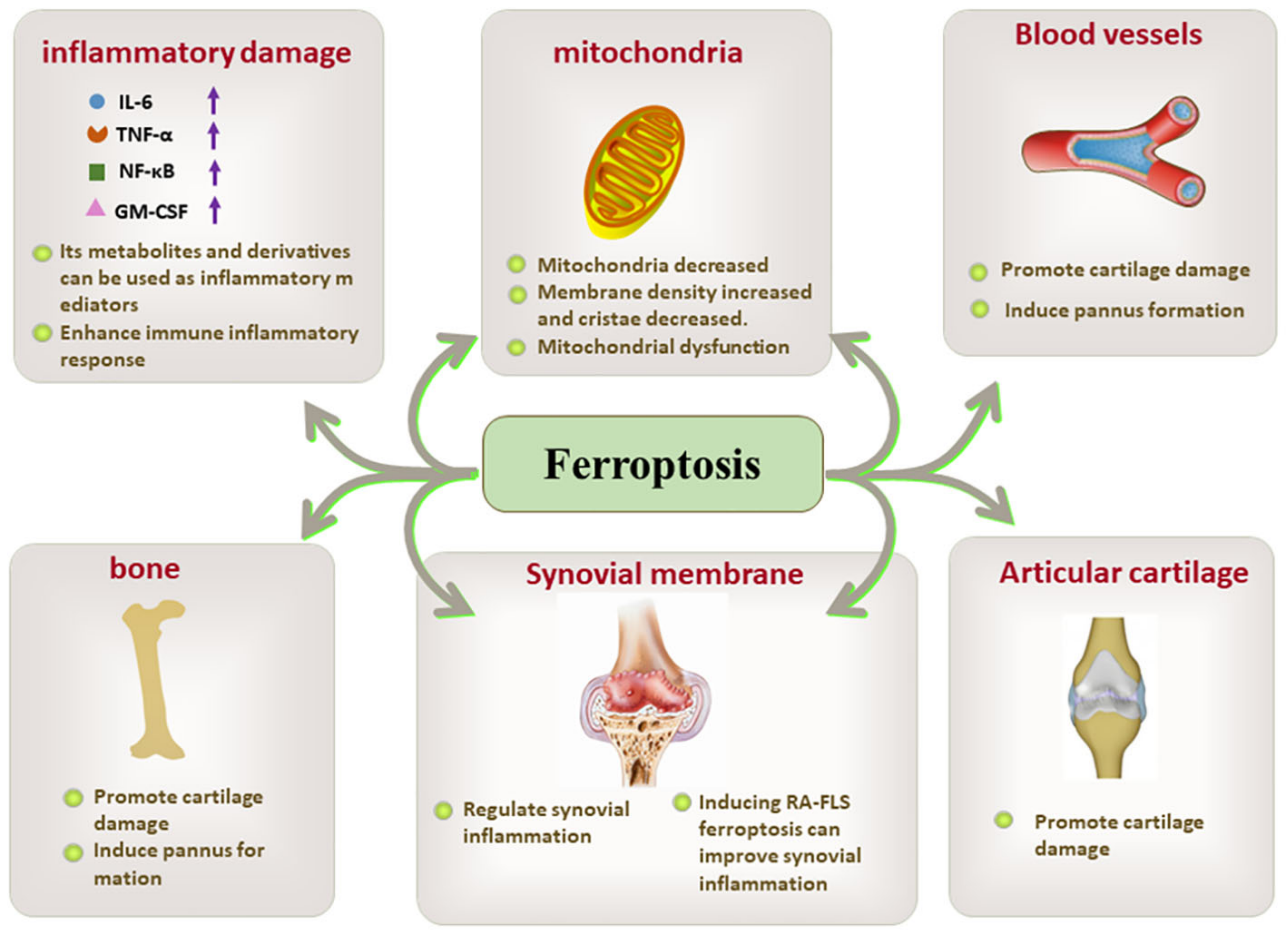
The key role of ferroptosis in inflammatory damage, mitochondria, blood vessels. bone, synovial membrane, and articular cartilage.

PUFAs are responsible for ferroptosis-inducing lipid peroxidation. PUFA-derived lipid mediators, AA and docosahexaenoic acid (DHA), serve as inflammatory activators in RA (79). COX-2, an enzyme involved in the AA oxidation reaction, rapidly induces inflammatory responses at the site of inflammation and accelerates the inflammatory process under specific conditions. In the presence of PPARγ (peroxisome proliferator-activated receptor γ)-dependent mechanisms, the expressions of COX-2 and prostaglandin D synthase 2 (PGD2) is promptly upregulated, resulting in a vicious cycle that enhances the inflammatory response in a positive feedback manner (80, 81). Although the role of ferroptosis and associated inflammatory mediators in RA has been supported to some extent, further in-depth scientific studies are still necessary to fully understand this complex process (**Figure 3**).

### 5.2 Ferroptosis regulates RA synovial inflammation

**Figure 4:**
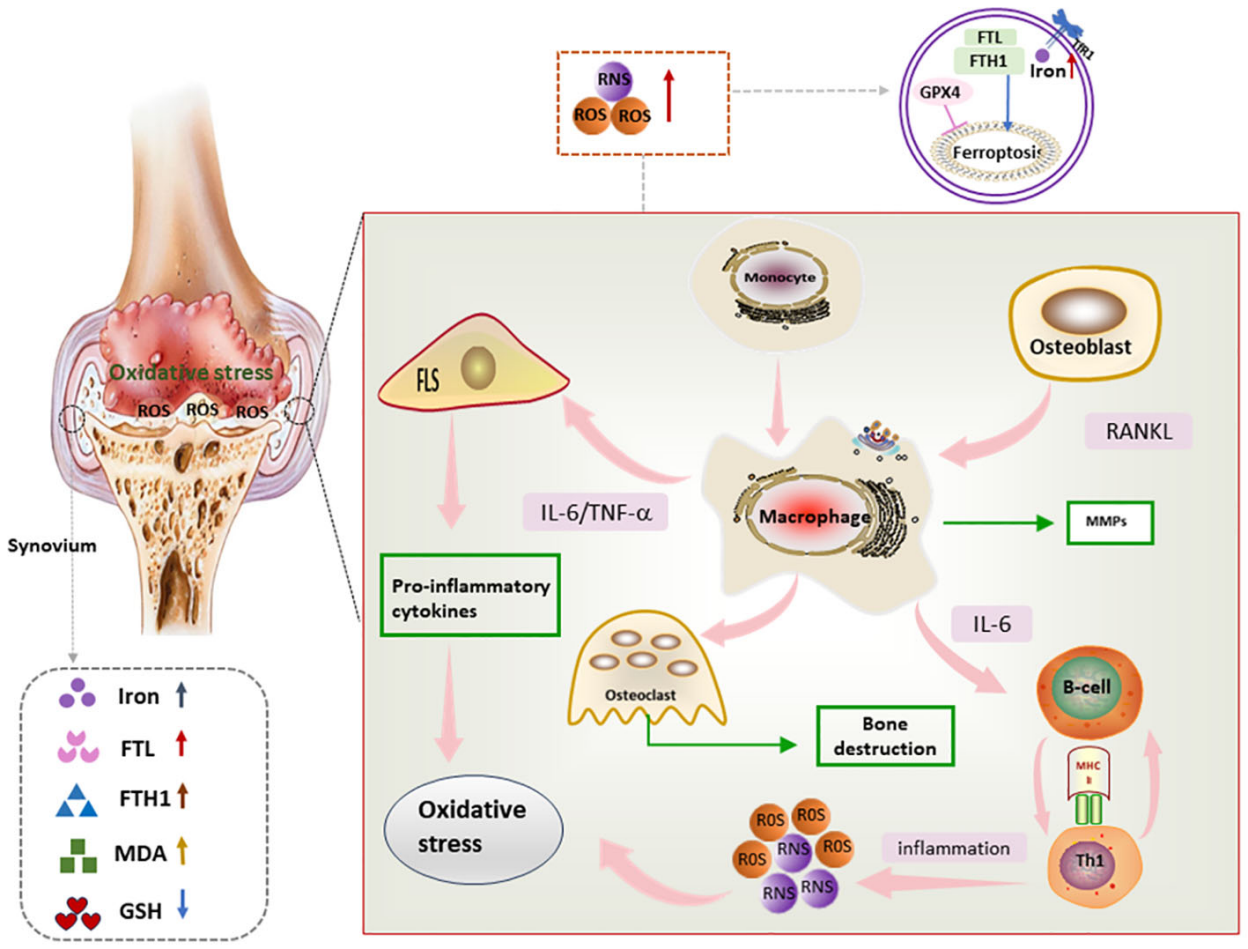
Mechanism of ferroptosis in RA synovium Lipid peroxides as well as iron deposition can be seen in RA synovium. In addition, proinflammatory factors secreted by immune cells and FLS in RA synovium promote oxidative stress, which in turn further exacerbates the inflammatory response, and through this vicious circle, ferroptosis and synovial inflammation are promoted, FLS, fibroblast-like synoviocyte; IL-6, interleukin 6; RNS, reactive nitrogen species; ROS, reactive oxygen species; TNF, tumor necrosis factor; RANKL, Receptor Activator for Nuclear Factor-kB Ligand; MMPs, matrix metalloproteinases.

Synovial inflammation is a central event in the pathogenesis of RA, as well as a major trigger for bone and cartilage destruction and vascular opacification formation. Synovium is mainly composed of FLSs and immune cells. FLSs, the dominant non-immune cells of the synovium, develop abnormal proliferative capacity and aggressive characteristics under oxidative stress conditions (82, 83). Immune cells including macrophages, lymphocytes, and T cells play immune surveillance and inflammatory regulatory roles in the synovium. Activated T cells and macrophages in the synovium form an environment conducive to ferroptosis and inflammation, and also induce ROS production in a positive feedback manner, thus promoting the release of pro-inflammatory factors into the synovium and synovial fluid and further amplifying synovial inflammation (84). Moreover, compared with osteoarthritis, both RA synovial tissues and synovial fluids present ferroptosis-related characteristics such as lipid peroxidation and iron overload, as well as massive cascading proliferation and “tumor-like” growth of RA-FLSs (85) (**Figure 4**).

FLSs are considered to be dominant drivers of RA pathogenesis. Fibroblast activating protein-α (FAPα) is a RA fibroblast marker located on the synovial cell surface. According to a previous study (85), the number of FAPα^+^ fibroblasts is significantly increased in the inflammatory synovium of collagen-induced arthritis (CIA) mice. The TNF antagonist etanercept can significantly enhance the killing effect of the ferroptosis inducer imidazole ketone erastin (IKE) on RA-FLSs, and the combination of the two restores synovial homeostasis by promoting FAPα^+^ fibroblasts ferroptosis and reducing the number of FAPα^+^ fibroblasts. Thus, inducing the ferroptosis of RA-FLSs may be a potential target for the treatment of RA. In contrast, treatment with the GPX4 inhibitor RSL3 is observed to specifically induce cell death in FAPα^+^ fibroblasts. Interestingly, RSL3 does not increase cell death in macrophages, endothelial cells, T cells, or B cells (85). The possible reasons for this may be as follows. Firstly, RA-FLSs proliferate abnormally in response to the elevation of ROS and lipid oxidation, and the GPX4 inhibitor RSL3 specifically scavenges ROS accumulation in the synovial membrane and downregulates the number of FLSs. Therefore, FLSs are sensitive to RSL3. Secondly, FAPa^+^ fibroblasts are almost undetectable in non-inflammatory conditions, while they significantly increase in inflamed synovial membranes, and the synovial inflammatory milieu tends to promote ferroptosis and lipid ROS production, and induction of ferroptosis is effective in reducing the number of FLSs and thereby improving RA. It can thus be concluded that GPX4 inhibitors have the potential to selectively affect the cell survival in specific cell types. However, further studies are still needed to comprehensively validate and understand the therapeutic potential of GPX4 inhibitors and their effects on different cell types. Not only that, future studies are expected to identify surface proteins more specific to fibroblasts, which will facilitate the development of fibroblast ferroptosis targeting therapy.

Glycolysis is an essential energy production and metabolic process, and its regulatory mechanism involves several signaling pathways such as Akt/mTOR/HIF-1α (86). Overexpression of SLC2A3 activates glycolysis and HIF-1 signaling pathways (87). In the meantime, the ferroptosis inducer RSL3 can trigger ferroptosis of RA-FLSs by reducing the expression of SLC2A3 and suppressing the glycolytic metabolism of RA-FLSs, which further reveals the intrinsic mechanism of RSL3-induced ferroptosis of RA-FLSs.

The nuclear factor E2-related factor 2 (Nrf2)-related anti-oxidative stress is strongly associated with ferroptosis suppression. Nrf2 activation can prevent oxidative stress and inflammatory damage in RA synovium by transcribing various antioxidant enzymes including superoxide dismutase (SOD), heme oxygenase-1 (HO-1), and GSH (88). It is discovered (89) that activation of Nrf2 not only regulates a series of inflammation-related signaling molecules but also inhibits the production of ROS and represses the proliferation and migration of RA-FLSs, thereby effectively alleviating RA synovial inflammation.

### 5.3 Ferroptosis exacerbates bone and cartilage damage in RA

In addition to synovial inflammation, progressive bone and cartilage destruction is another catastrophic pathological change in RA. The imbalance between bone resorption by osteoclasts and bone formation by osteoblasts is the major contributor to the development of bone damage in RA, and ferroptosis further exacerbates this process.

Osteoclasts are multinucleated giant cells formed by the fusion of monocyte/macrophage precursor cells. M-CSF and RANKL are crucial for osteoclast differentiation and development. A typical osteoclast phenotype on the surface of multinucleated giant cells can be observed at the bone-pannus joint interface in CIA rats, suggesting the involvement of osteoclasts in RA-associated bone destruction (90). Furthermore, cytokines such as IL-17 and IL-6 in RA arthritis induce RANKL expression in osteoblasts and RA-FLSs, indirectly stimulating osteoclast formation (91, 92). ROS, a principal feature of ferroptosis, also interacts with RNAKL and M-CSF to promote osteoclast differentiation. In addition, activation of the Janus kinase (JAK)2/STAT3 pathway effectively promotes RANKL expression and induces osteoclast differentiation, and ROS can activate the JAK2/STAT3 pathway by upregulating HIF-1α expression (49, 52). An interesting experiment shows (93) that iron overload induces osteoblast apoptosis and osteoclast differentiation, and that icariin can inhibit the function of osteoclasts by regulating ROS and mitochondrial membrane potential (MMP) homeostasis while attenuating iron overload-induced oxidative damage in osteoblasts.

As articular cartilage is an important structural and functional unit of the joint, cartilage degeneration and damage can cause impairment and loss of joint function in RA patients (94). On the one hand, IL-7 and TNF in RA can degrade cartilage by stimulating chondrocytes to secrete cartilage-degrading metalloproteinases (MMP). On the other hand, the enhanced sensitivity of chondrocytes to IL-1 and TNF in synovial fluid in turn accelerates cartilage degradation (95). Not merely that, knockdown of GPX4 is found to increase chondrocyte sensitivity to oxidative stress and also activate the MAPK/NF-κB pathway to promote extracellular matrix (ECM) degradation, while this process can be effectively mitigated by the use of Fer-1, a ferroptosis inhibitor (96). However, the research on ferroptosis and bone and cartilage damage in RA is still in its infancy and still needs more supporting evidence.

### 5.4 Effect of ferroptosis on osteoporosis

RA patients have both disease-specific risk factors for OP and fractures. OP is a common clinical disorder mainly characterized by bone mass loss and bone microarchitectural deterioration (97). During the course of RA, inflammatory factors induce osteoclast differentiation but inhibit osteoblast maturation. The aggravation of bone loss caused by this imbalance in bone metabolism is the main reason for RA complicated with OP (98, 99). Moreover, the long-term clinical use of mainstream drugs such as glucocorticoids and immunosuppressants can also induce the development of OP. Epidemiological surveys have shown (100) that approximately 60-80% of RA patients are complicated with OP.

Iron metabolism disorder is identified as an independent risk factor for OP. Iron overload can disrupt bone homeostasis by significant inhibition of osteoblast differentiation and stimulation of osteoclastogenesis, consequently leading to OP (101). In an iron overload rat model constructed by intraperitoneal injection of iron dextrose, iron overload causes thinning of bone trabeculae and cortex, and increase of bone resorption. Further findings (102) reveal that iron overload leads to the increase of MMP and the accumulation of lipid peroxide by affecting GSH and fatty acid cycle, which further promotes the activation of osteoclasts and apoptosis of osteoblasts, leading to increased bone resorption and decreased bone formation.

Furthermore, the hypoxic microenvironment of RA is known to inhibit RANKL-induced ferritin phagocytosis and protect osteoclasts from ferroptosis. Conversely, the HIF-1α inhibitor 2ME2 effectively promotes osteoclast ferroptosis and prevents ovariectomy-induced OP in rats (52). Not only that, targeting osteoblast ferroptosis is also a potential therapeutic strategy for OP. One study has found (103) that the expression of NADPH oxidase 4 (NOX4) is elevated in OP mice and patients, and NOX4 overexpression drives osteoblast ferroptosis and subsequently causes bone loss in OP mice.

### 5.5 Ferroptosis modulates RA pannus formation and angiogenesis

Pannus formation in the synovial cavity is a major pathological factor in RA and can cause irreversible damage to the joint and cartilage. Synovial pannus is composed of neoplastic microvessels, proliferating hypertrophic synovial cells, inflammatory cells, and mechanized fibrin, exhibiting tumor-like tissue properties (104). Angiogenesis is an early event in the pathogenesis of RA, which is crucial for the proliferation of synovial tissue and the formation of pannus (105, 106).

RA-FLSs exhibit tumor-like biological characteristics that facilitate pannus formation. On the one hand, RA-FLSs secrete reactive substances, such as IL-6, MMP, and TNF-α, which not only aggravate the process of bone erosion but also exacerbate the development of pannus (107). On the other hand, FLSs produce MMP to digest a variety of proteins in cartilage and supporting structures, further promoting the invasion and expansion of pannus (108). In addition, VEGF secreted by RA-FLSs is an important factor in promoting angiogenesis (109, 110). Thus, it is evident that inducing ferroptosis of RA-FLSs not only regulates synovial homeostasis but also has important implications for the inhibition of angiogenesis.

An appropriate ROS concentration is conducive to normal angiogenesis and vascular homeostasis, while a high concentration of ROS chronically induces ferroptosis and consequently causes pathological damage to blood vessels. However, it must be emphasized that ROS as a trigger for RA and FLS proliferation is not an inevitable consequence. A recent study (111) has indicated the involvement of ROS in VEGF-dependent signaling processes in endothelial cells. Specifically, ROS transcribes VEGF genes by activating the NF-κB pathway to participate in key pathological processes such as angiogenesis and proliferation. In addition, ROS also functions in other cell types, such as macrophages, fibroblasts, endothelial cells, and keratin-forming cells. These cells produce VEGF in response to ROS stimulation and regulate angiogenesis through upstream and downstream effects of VEGF/VEGFR2 signaling (112, 113).

The Notch signaling pathway responds to the regulation of ROS and is closely associated with RA angiogenesis (53). Its mechanism may be related to the induction of angiogenesis and endothelial cell migration by activating FLSs. Due to insufficient research results, it is necessary to further explore the relationship between ferroptosis and RA angiogenesis, which may help to reveal new mechanisms underlying the pathological process of RA and provide potential targets for the development of new therapeutic strategies.

## 6 Ferroptosis may be a key target in controlling RA

Compelling evidence supports the potential of ferroptosis as a prospective target for the prevention and treatment of RA (114–116): using ferroptosis inducers to induce ferroptosis of RA-FLSs or using ferroptosis inhibitors to reduce inflammation and joint damage. Searching for ferroptosis inhibitors or inducers has become a hot research topic in recent years, but due to the intricate regulatory mechanisms of ferroptosis and individual differences of patients, the targeted therapy of ferroptosis still stays in the preliminary stage.

Consistent with previous findings that promoting ferroptosis of RA-FLSs improves RA, the results of a recent study also indicate (117) that glycine drives ferroptosis of RA-FLSs through methylation of the S-adenosylmethionine (SAM)-associated GPX4 promoter and further enhances this effect by reducing FTH1 expression. This study provides important insights into the molecular mechanisms of ferroptosis in RA and the development of new therapeutic strategies. However, further studies are still needed to validate and extend these results.

Another study has also found (118) that the bioactive peptide G1dP3 promotes RA-FLS ferroptosis via a p53/SLC7A11 axis-dependent manner and has a potential therapeutic role in RA. It is speculated that somatic mutations of p53 in RA-FLSs may be the direct cause of synovial hyperplasia and subsequent pannus formation (119). p53 is one of the most commonly mutated tumor suppressor genes, and p53 expression is elevated in activated FLSs (120). Studies have confirmed (121, 122) that p53 can induce ferroptosis not only by inhibiting SLC7A11 activity but also by increasing CDKN1A expression or decreasing DPP4 activity, suggesting the dual regulatory role of p53 in the regulation of ferroptosis. In addition, ferroptosis induced by salazosulfapyridine likewise exerts dual effects (123). On the one hand, salazosulfapyridine inhibits ferroptosis by inhibiting systemic Xc^-^ and downregulating GSH and GPX4. On the other hand, salazosulfapyridine induces the Fenton reaction by upregulating the ferric ion levels, thus generating excessive lipid ROS and inducing ferroptosis.

Antioxidants also have a regulatory effect on ferroptosis. ROS promotes Th17 differentiation and IL-17 production through activation of the RORγt and STAT3 pathways. CoQ10, a ROS scavenger and anti-inflammatory substance, has been shown to regulate Th17 and IL-17 through the STAT3 pathway, thereby inhibiting ferroptosis and ameliorating joint inflammation in CIA mice (124). In addition, the antioxidant and anti-inflammatory effects of natural extract polyphenols (like resveratrol, quercetin, rutin, curcumin, etc.) have been widely reported (125). Sirtuin 1 (SIRT1) prevents inflammatory responses in articular chondrocytes, and resveratrol, a natural SIRT1 activator, can exert a protective effect on chondrocytes by inhibiting NF-κB and MMP-13 expression (126). Green tea polyphenol EGCG has been shown to activate the antioxidant defense system in chondrocytes by inhibiting ERK and p12 MAPK activation while blocking pro-inflammatory signaling pathways, thereby alleviating RA (127). Xanthine oxidase (XO) is an essential generator of free radicals, and quercetin has been proven to stifle XO and prevent oxidative damage. In addition, one study reports that icariin can inhibit ferroptosis of synovial cells and exert protective effects by activating the Xc-/GPX4 axis (128).

As described in a previous study (129), TRPM7-mediated ferroptosis of chondrocytes is likely to be a promising target for the prevention and treatment of RA. TRPM7 is highly expressed in the articular cartilage of adjuvant arthritis rats and knockdown of TRPM7 alleviates articular cartilage destruction in RA (130). Further, inhibition of TRPM7 activity attenuates RA articular cartilage destruction and chondrocyte ferroptosis by suppressing the PKCα-NOX4 axis (129). Moreover, another study has discovered (131) that glycyrrhizin, a natural flavonoid extracted from Ural licorice root, can effectively ameliorate RA inflammation by blocking the MAPK signaling and inhibiting angiogenesis.

Despite the promising results on ferroptosis modulators revealed by preclinical studies, the effects of sustained ferroptosis modulation on normal tissues and organs remain unresolved. Therefore, it is essential to conduct comprehensive toxicity studies and monitor potential off-target effects to ensure the safety of ferroptosis modulation in RA treatment.

## 7 Discussion

Ferroptosis can be utilized as an interventional target for RA treatment. However, the therapeutic effects of ferroptosis regulation may vary from tissue to site since ferroptosis is an extremely complex process. For instance, although ferroptosis inducers can effectively suppress RA synovial inflammation by inducing FLSs ferroptosis, the use of ferroptosis inducers may also exacerbate inflammatory responses given the pro-inflammatory nature of ferroptosis, thus promoting bone erosion and bone destruction in RA, and further inducing OP development and angiogenesis. Specifically, two issues need to be considered for the targeted therapy of ferroptosis in RA. One is the off-target effect of non-FLS cell types, as ferroptosis induction may affect other cell populations in the synovium. Another consideration is the delicate balance between promoting ferroptosis of target cells and minimizing excessive cell death, which may lead to tissue damage and healing impairment. Therefore, the underlying mechanisms of ferroptosis events and autoimmune diseases still need more scientific exploration, and further studies are required to optimize the specificity and efficacy of ferroptosis-targeted drugs in RA.

Except for ferroptosis, there are other cell death modalities in RA, such as apoptosis, pyroptosis, autophagy, and necrosis, which coexist in a mixed form and play a synergistic role in the pathological progression of RA. The interactions and regulatory dynamics among these cell death pathways are not fully understood. Further research on the interactions and specific functions of these cell death pathways in RA contributes to a deeper understanding of the pathological mechanisms and targeted therapeutic strategies of RA. In addition, the current understanding remains limited regarding the cellular mechanisms of different cell death isoforms to maintain the stability of the internal environment, respond to external stresses, and defend against pathogenic threats. There are a lot of unknown aspects in this field that require further in-depth studies.

Furthermore, no consensus has been reached regarding the potential deleterious or protective effects of ferroptosis in RA. A key question that remains to be addressed is the identification of the optimal window for modulating ferroptosis and achieving therapeutic balance. In particular, the optimal timing and duration of ferroptosis induction in RA-FLSs are unclear. How to select and when to apply the best inducer or inhibitor applicable to ferroptosis in RA also demands in-depth studies. Another critical issue is the lack of specific biomarkers for monitoring ferroptosis activity in RA. The development of reliable biomarkers not only contributes to patient stratification and treatment response monitoring, but also helps to assess the efficacy of iron ferroptosis-targeted therapies in clinical trials.

In conclusion, ferroptosis in RA represents an exciting research field with important research and application prospects. in-depth research on the regulatory mechanisms of ferroptosis and the role of ferroptosis in RA is expected to lead to the development of more precise and efficient ferroptosis-targeted therapeutic strategies for RA.

## Author contributions

HuZ: Writing – original draft. CT: Writing – review & editing. MW: Writing – review & editing. HoZ: Writing – original draft. YZ: Writing – review & editing.
